# Towards a harmonized testing strategy for nanofibers by integrating toxicological screening and proteomic profiling

**DOI:** 10.1038/s41598-025-15423-9

**Published:** 2025-09-12

**Authors:** Rico Ledwith, Verónica I. Dumit, Tobias Stobernack, Victor Alcolea-Rodriguez, Antje Bergert, Doreen Wittke, Andrea Haase, Mario Pink

**Affiliations:** 1 Department of Chemical and Product Safety, German Federal Institute for Risk Assessment (BfR), Berlin, Germany; 2 Institute of Pharmacy, Freie Universität Berlin, Berlin, Germany; 3 Spanish National Research Council (CSIC), Institute for Catalysis and Petroleum Chemistry, Madrid, Spain

**Keywords:** Nanosafety, New approach methodology (NAM), Carbon nanomaterials, Multiwalled carbon nanotubes, Fiber pathogenicity paradigm (FPP), THP-1 cells, Biochemistry, Molecular biology, Systems biology, Biomarkers, Risk factors

## Abstract

Nanofibers, particularly multi-walled carbon nanotubes, have attracted attention for their exceptional properties, but concerns remain about their potential health hazards due to their fiber-like morphology. Although bio-durable nanofibers may cause cancer upon inhalation, only rigid nanofibers may exhibit morphology-driven pathogenicity. Since no validated methods exist for assessing their rigidity, alternative approaches are needed that comply with the 3R principles (Replacement, Reduction, Refinement) and the European Commission efforts to foster alternatives to animal testing. This study aims to advance the development of a harmonized test method for nanofibers toxicity by comparing effects of selected carbon-based nanomaterials (NMs) with different morphologies: a nanofiber (Mitsui-7-JRCNM40011a), an elongated material (NM-400) and a particle (Printex-90). Therefore, in vitro toxicological screening and proteomic investigations were employed using differentiated THP-1 (dTHP-1) macrophage-like cells. First, we evaluated cytotoxicity and pro-inflammatory responses of the different dTHP-1 phenotypes (M0, M1 and M2) to evaluate their sensitivity, and thus selected the M0 phenotype for further oxidative and lysosomal investigations: Mitsui-7-JRCNM40011a caused, besides increased cytotoxicity and pro-inflammatory effects, oxidative stress and lysosomal dysfunction. Moreover, decreased levels of 25 lysosomal proteins, including five cathepsins, were detected. These findings deepen the understanding of nanofiber-related toxicity, supporting the development of a reliable in vitro testing strategy.

**Supplementary Information:**

The online version contains supplementary material available at 10.1038/s41598-025-15423-9.

## Introduction

Nanofibers (NFs) are characterized by having at least two external dimensions in the nanoscale and have emerged as a focal point for research and development^1^. Multi-walled carbon nanotubes (MWCNTs), which are among the most extensively studied NFs, exhibit a variety of valuable properties, including thermal and electrical conductivity, making them advantageous for many industrial applications, such as in high performance batteries and medical biosensors^2–4^. However, their potential health hazards remain a subject of concern, particularly due to their morphological similarities to asbestos fibers, known to cause fibrosis, cancer and mesothelioma^5,6^. Understanding the potential of NFs to cause asbestos-like pathogenicity upon inhalation is crucial to enable the responsible usage of such materials.

The fiber pathogenicity paradigm (FPP) outlines that the morphology and bio-durability of fibers can potentially lead to asbestos-like lung diseases^7,8^ like fibrosis, cancer and pleural mesothelioma, an aggressive form of cancer almost uniquely associated with asbestos exposure. The World Health Organization (WHO) outlined for occupational settings counting criteria for fibers with potentially carcinogenic properties. These criteria stipulate that fibers exceeding lengths > 5 μm, with a respirable diameter < 3 μm, and an aspect ratio > 3:1, pose significant risks to human health upon inhalation exposure if they also meet the additional criterion of being sufficiently bio-durable^9^. However, NFs represent a challenge to the FPP. As the diameter of a fiber decreases, the fiber gains flexibility and becomes more prone to tangling^10^. Studies with thin MWCNTs indicate that those with diameters below 30 nm (the proposed bending threshold for MWCNTs^11^ tend to form spherical agglomerates, losing their fibrous nature^12^. The toxicity of entangled MWCNTs would align more closely with bio-persistent granular particles^13^.

Risk assessment of the pathogenicity of fibers currently relies on data obtained from animal testing, which is time-consuming and raises both ethical and scientific concerns. In addition, the European Commission is making efforts to phase out animal testing in the area of chemical safety assessment^14^. The challenges when using rodents for the assessment of fiber toxicity relate to the long latency periods for tumor development, which occur toward the end of their lifespan when they are susceptible to spontaneous tumor development. This makes it difficult to determine the true extent of the carcinogenic properties of NFs^15^. Therefore, to ensure the safe and sustainable use of rapidly emerging novel NFs, there is an urgent need for alternative assessment strategies, e.g., in form of new approach methodologies (NAMs), in particularly high throughput in vitro assays. However, so far only a few NAMs are validated, established as OECD test guidelines (TGs), and accepted for regulatory risk assessments. Existing NAM-based OECD TGs cover for example, skin corrosion/irritation and serious eye damage/irritation, which are acute effects associated with substance toxicity^16^. However, when considering NF toxicity, long-term effects addressing potential outcomes like fibrosis and cancer become essential.

To this end, the Adverse Outcome Pathways (AOPs) serves as a framework that allows to compile existing knowledge to understand long-term toxicological effects. Therefore, the AOPs are a crucial foundation for the design of testing strategies. AOPs link the molecular initiating event (MIE) to the adverse outcome through a well-defined sequence of key events (KEs) across molecular, cellular, organ, and organism levels, based on the current knowledge^17,18^. Each KE represents a measurable biological change^19,20^. Specifically for fiber toxicity, multiple AOPs, such as AOP171, AOP303, and AOP409, have been developed. Common to these AOPs is the induction of inflammation, with the macrophage response to fiber exposure playing a critical role in initiating the inflammatory cascade^20,21^. In particular, when alveolar macrophages encounter rigid fibers of critical lengths, the fundamental mechanism for particle clearance, the phagocytosis by the macrophage, is compromised, leading to a state known as frustrated phagocytosis^22,23^. The inability to successfully phagocytose large fibers triggers a cascade of detrimental effects, ultimately leading to cell death. These effects are accompanied by lysosomal disruption^24^ the release and generation of reactive oxygen species (ROS) and pro-inflammatory mediators^25^, which recruit additional immune cells, such as circulating monocytes^23^. Following extravasation, monocytes are maturated in the tissue to non-activated (M0) macrophages, which are further polarized by local stimuli to type 1 (M1) or type 2 (M2) macrophages^26^. M1 macrophages are found promptly at the affected site to release pro-inflammatory cytokines (inflammatory macrophages), while M2 macrophages appear later to counteract the pro-inflammatory activity of the M1 phenotype by secreting anti-inflammatory mediators (anti-inflammatory macrophages).

Differentiated human monocytic THP-1 macrophage-like cells (dTHP-1) are a prominent cell model frequently used for studying macrophage responses to fibers owing to a very limited number of better alternatives^27,28^. Compared to primary human monocyte-derived macrophages, which are short-lived, finite, and have high inter-doner variability, dTHP-1 cells are more robust cell model that might be easier to standardize^29^. Moreover, they are widely used for studying immunology, infectious, and inflammatory diseases, among others^30–32^. Also, they are utilized in the Human Cell Line Activation Test (h-CLAT), a component of one of the limited number of NAM-based OECD test guidelines^33^. THP-1 cells can be readily differentiated into the M0 phenotype, and further polarized into the M1 and M2 macrophage phenotypes, offering versatile possibilities for toxicity studies and appearing well suited for use in a harmonized fiber testing strategy. While previous research mainly focused on M0 macrophages, recent endeavors have incorporated M1 and M2 phenotypes^28,34^. However, there is considerable variation in the experimental conditions used for differentiation and polarization^35–37^. Therefore, a crucial starting point for establishing the testing procedure is to select the most appropriate macrophage phenotype. The selected dTHP-1 phenotype must provide the most sensitive readouts for NF exposure, particularly to the varying rigidity of the NFs, as it is imperative to extend the FPP to encompass this parameter^10^. The lack of validated methods for assessing NF rigidity presents a significant challenge. Therefore, different approaches are required, such as investigating the effects of NFs on cellular responses.

In a previous study, we conducted a meta-analysis of over one hundred existing omics datasets from both human and rodent in vitro and in vivo data, demonstrating the potential of this approach of identifying relevant signatures for different morphologies of carbon-based NMs, including entangled and rigid NFs^38^. Although this information contributes to the development of a harmonized fiber testing strategy, distinct responses to the various morphologies of the evaluated carbon-based materials were observed at the level of cellular pathways^38^. No specific genes or proteins were identified as potential biomarkers. To deepen the mechanistic understanding of NF toxicity, and aiming at advancing the development of a harmonized testing strategies for NFs, in this work we investigated the suitability of different dTHP-1 phenotypes. To this end, we assessed the cytotoxicity and cellular responses towards Printex-90, NM-400, and Mitsui-7-JRCNM40011a—representing a spherical particle, an entangled fiber, and a carcinogenic rigid fiber, respectively. The assays address cytotoxicity, pro-inflammatory cytokine release, and oxidative stress and lysosomal integrity. To further unravel the underlying cellular toxicity mechanisms caused by the morphologically different carbon NMs, we additionally employed proteomic profiling using an advanced tandem mass tag (TMT) labelling protocol^39^. Our findings highlighting the inherent limitations of certain dTHP-1 cell models for comprehensively evaluating fiber toxicity, and provide a molecular description of the morphological-driven toxicity for Mitsui-7-JRCNM40011a.

## Materials & methods

### Nanomaterials

The NMs investigated in this work and their characteristics are outline in Table 1. NMs were dispersed by sonication following the NANoREG D2.08 SOP 02 standard operational procedure^40^. According to the protocol, a 2.56 mg/mL stock dispersion is prepared by prewetting the NMs with 0.5 vol% ethanol, followed by dispersion in 0.05 wt% BSA-water. Stock dispersion are prepared to relative exposure concentrations in complete cell culture medium containing 10% fetal calf serum (FCS). All NMs were shown to be endotoxin-free using Limulus Amebocyte Lysate Endochrome test (Lonza, Switzerland, 11690231).

**Table 1:** NMs and their characteristics used in the present study.

MWCNT	Manufacturer Provider	Mean Length	Synthesis	Mean Thickness (nm)	BET (m^2^/g)	Main Impurities	Shape
Printex-90^41^ Lot:237,246	Orion (Degussa), Germany. *	14 nm	Furnace	14	300	Organic impurity content is < 1%. 0.8% N and 0.01% H_2_	Spherical particle
NM-400^42^ Lot: JRCNM-400a	JRC, Italy.	846 ± 446 nm	Chemical vapor deposition	11 ± 3	254	> 0.01%: Al, Fe, Na, S 0.005–0.01%: Co 0.001–0.005%: Ca, K	Highly bent (entangled agglomerates)
Mitsui-7^43^ Lot:05072001K28	Mitsui & Co., Japan. *	3.86 ± 1.94 μm #	Chemical vapor deposition	49 ± 13.4	26	0.41% Na, 0.32% Fe	Rigid wall (needle-like)

^#^The measured values given are mean values, originated from Porter et al. 2010^43^. However, the decisive factor is the consideration of the entire length distribution, which contains a proportion of fibers with lengths > 5 μm, as indicated by Takagi et al. 2008^44^. *Distributed by the European Commission-DG JRC.

### Cell lines and culture conditions

**THP-1** (DSMZ, ACC 16): The human monocytic leukemia cell line was obtained from the German Collection of Microorganisms and Cell Cultures GmbH, Braunschweig, Germany. THP-1 monocytes were maintained in culture in Roswell Park Memorial Institute medium (RPMI 1640), (PAN Biotech, Germany, P04-17500), 10% heat inactivated FCS, (PAN Biotech, Germany, P30-3302) and supplemented with 1% penicillin-streptomycin, (PAN Biotech, Germany, P06-07100), 1% L-glutamine, (PAN Biotech, Germany, P04-80100), 10 mM HEPES buffer, (PAN Biotech, Germany, P05-01100) and 1mM sodium pyruvate, (PAN Biotech, Germany, P04-43100). This is then referred to as complete cell culture medium (CCM). The range of the passage number used for experiments, P6 - P13, was deemed reliable for conducting reproducible biological replicates for proteomic experiments as previously assessed^45^. Cell lines were confirmed to be mycoplasma free and were incubated at 37 °C, 5% CO_2_. The cells were split twice per week in T75 flasks (TPP, Switzerland, 90075), to 1 × 10^6^ cells in 15 mL CCM.

All plasticware used in this study was sourced from TPP Switzerland.

### THP-1 differentiation conditions to obtain initial M0, M1 and M2 phenotypes

THP-1 cells were differentiated to M0 macrophages by incubation with 100 nM phorbol-12-myristate-13-acetate (PMA), (Sigma Aldrich, Germany, P1585) in CCM for 48 h. To obtain both M1 and M2 macrophages, the initial PMA differentiation time to the non-activated (M0) state was shortened to 24 h. Based on Mirata et al.,^28^, the M0 dTHP-1 macrophages were incubated with 20 ng/mL of IFN-γ (Peprotech, Germany, 300-02) and 200 ng/mL of Lipopolysaccharide (LPS), (Sigma Aldrich, Germany, 297-473-0) in CCM for 24 h to obtain polarized M1 macrophages and with 20 ng/mL of interleukin 4 (IL-4), (Peprotech, Germany, 200-04) in CCM for 24 h to obtain polarized in M2 macrophages.

### Cell plating and exposure to NFs

For all cell-based assays, 2 × 10^4^ THP-1 cells in 100 µL CCM were plated in 96-well plates, unless otherwise stated, and subsequently differentiated into various dTHP-1 phenotypes. No resting period was used before further polarization. After differentiation, the adherent cells were washed three times with Dulbecco’s Phosphate Buffered Saline (DPBS), (PAN Biotech, Germany, P04-36500) and then directly exposed to NMs or positive controls suspended in 100 µL of CCM for 24 h. As interference controls, cell-free wells were similarly exposed to NMs. For assays using cell supernatants, the supernatants were first transferred to V-bottom 96-well plates and then centrifuged at 5,000 relative centrifugal force (RCF) for 10 min to pelletize any suspended NMs. Adherent cells used were washed three times with DPBS to remove NMs before incubation with the respective assay probes. All assays were performed in three independent replicates, unless otherwise stated.

### Cytotoxicity and metabolic activity: LDH and WST-1 assays

Both the lactate dehydrogenase (LDH) and the water-soluble tetrazolium (WST-1) assays were performed in tandem. Following cell exposure, 50 µL of the centrifuged supernatants were utilized for the LDH assay while the remaining adherent cells were utilized for the WST-1 assay.

LDH is released into the supernatant as a result of cell membrane permeabilization and this is well-known marker for the assessment of cell membrane integrity and cytotoxicity. The LDH release was quantified using a commercially available LDH diagnostic kit (Roche, Germany, 11644793001), according to the manufacturer’s protocol. Absorbance readings were taken at 490 nm with a reference wavelength of 630 nm. The measured absorbance values are normalized to the respective positive control on each 96 well plate. Positive control cells were treated with 1% of Triton-X 100 (applied to foster maximal cell permeabilization) for 30 min before the end of the 24 h exposure period to NFs.

The WST-1 assay is commonly used to measure cell viability and proliferation by means of metabolic conversion of the water-soluble tetrazolium salt to soluble formazan dye. The WST-1 dye (Roche, Germany, 5015944001), was diluted 1:10 in CCM according to the manufacturer’s protocol and then incubated with cells for 3 h. Absorbance readings were taken at 450 nm. The obtained values were normalized to the respective control wells on each well plate.

### Inflammation state of dTHP-1 cells: TNF-α release

TNF-α concentrations in supernatants were assessed using the Human TNF-α DuoSet ELISA; (RNDSystems, USA, DY210), according to the manufacturer’s protocol. Absorbance readings were taken at 450 nm with a reference wavelength of 570 nm. The absorbance values are normalized to a TNF-α standard curve and expressed as TNF-α released in pg/mL. 1 µg/mL of LPS exposed to cells for 24 h, is used as a pro-inflammatory positive control.

### Lysosomal integrity: neutral red assay

Lysosomal integrity was assessed by means of the Neutral Red (NR) assay. NR is a weak cationic dye, which accumulates in viable lysosomes due to the pH gradient (lysosomal pH ~ 4.5), where it becomes protonated and trapped. The assay was conducted according to the method of Repetto et al., 2008, with minor adaptations^46^. After exposure of the cells to the various NFs, they were incubated with 40 µg mL^− 1^ of the NR dye (Merk, Germany, 1013690100), diluted in CCM, for 2 h. The dye was extracted using 50% ethanol containing 1% (v/v) acetic acid and the absorbance was quantified at 540 nm^46^. The absorbance values are normalized to the respective control wells on each 96 well plate and expressed as percentages.

### Oxidative protein damage: carbonylation assay

The carbonyl-fluorometric assay kit (Cayman Chemical, USA, CAY701530), was used to measure the protein carbonyl content in cell lysates to estimate oxidative damage to proteins and is based on the rhodamine B hydrazide (RBH) reaction. 4.5 × 10^5^ THP-1 cells in 3 mL CCM were plated in 6-well plates before further differentiation. Cell lysates were prepared according to manufactures protocol. Formation of the fluorescent protein carbonyl-RBH hydrazone was analyzed using an excitation wavelength of 560 nm and an emission wavelength of 585 nm. The fluorescence values are normalized to a carbonyl standard curve and then expressed as a fold change relative to the control wells.

### Reactivity measurements: DCFH_2_-DA assay

The assay is a fluorometric tool for the detection of acellular ROS generation and is based on quantifying the oxidation of the non-fluorescent probe 2′,7′-dichlorodihydrofluorescein (DCFH_2_) by ROS to the intensely fluorescent 2′,7′-dichlorofluorescein (DCF). The assay has no specificity for any particular ROS, and was previously applied to assess reactivity of various NMs in dTHP-1 cells^47^. The assay was conducted according to the SOP published by Boyles et al., 2022^48^. 3-Morpholinosydnonimine hydrochloride (SIN-1 hydrochloride), (Abcam, UK, ab141525) was utilized as a positive control. Test materials and positive control are prepared in phenol red-free medium at various concentrations. A volume of 25 µL of each preparation was added to a black, clear-flat-bottom 96-well plate, followed by 225 µL of 50 µM DCFH_2_ in DPBS. The samples were immediately read using an excitation wavelength of 485 nm and emission wavelength of 530, and then again after 30, 60, and 90 min. Particle-induced ROS generation was calculated by subtracting time-zero values from each subsequent timepoint, and normalizing the arbitrary fluorescence values to molar values of oxidized fluorescein diacetate (F-DA) using a standard curve. Dose-dependent false-positive signals, resulting from interference of the test material interference, either due to auto-fluorescence or fluorescence quenching, were assessed by replacing DCFH_2_ with either DPBS or 0.1 µM F-DA, and reading the plate using the same excitation and emission settings.

### TMT – labelling

Cell lysis, protein extraction, and digestion were performed with PreOmics iST-NHS kit (PreOmics, Germany, P.O.00026) according to the manufacturer’s protocol. Cell lysates were labelled using the TMT duplex Isobaric Label Reagent Set (Thermo Fischer, Germany). A previously established bottom-up proteomic protocol by Stobernack et al., 2024 was employed for this purpose^39^. In brief TMT6-plex labels (80 µg/4.1 µL) were added to protein digest (10 µg/18 µL, 0.1 M triethylammonium bicarbonate), incubated (1 h, 300 rpm, room temperature) and quenched (1.5 µL 5% hydroxylamine). Peptides were acidified (220 µL, 0.1% formic acid), cleaned up using the PreOmics iST-NHS kit, pooled (1:1 ratio), dried under vacuum (40 °C, 2.5 h), and reconstituted (0.1% trifluoroacetic acid (TFA), 5% acetonitrile) to 50 ng µL⁻¹ for LC-MS analysis.

### MS sample preparation, liquid Chromatography–Electrospray Ionization–Tandem mass spectrometry (LC–ESI–MS/MS) measurements

Cell pellets were prepared for proteomics measurements using the iST kit (PreOmics, Germany, P.O.00030) to obtain a tryptic peptide digest. Desalted peptides were reconstituted in 0.1% (v/v) TFA, 5% (v/v) acetonitrile to a final concentration of 50 ng/µL and transferred to vials with glass inserts. LC-MS analyses were performed on an UltiMate 3000 RLSCnano system (Thermo Scientific, USA) connected to an Orbitrap QExactivePlus (Thermo Scientific, USA) mass spectrometer. The LC system was coupled to the mass spectrometer via a nanospray flex ion source equipped with a stainless-steel emitter (Thermo Scientific, USA). Samples were injected (250 µg) and concentrated on an Acclaim PepMap100 C18 trap column (3 μm, 100 Å, 75 μm i.d. × 2 cm, Thermo Scientific, USA) equilibrated (5 µL/min, 5 min, 45 °C) with 0.05% TFA, 2% acetonitrile in water. After switching the trap column inline, peptides were separated on an Acclaim PepMap100 C18 column (2 μm, 100 Å, 75 µmi.d. × 25 cm, Thermo Scientific) at an eluent flow rate of 0.3 µL/min using a two linear gradient (5 to 35% B in 90 min, 35 to 50% in 5 min). Mobile phase A contained 0.1% formic acid in water, and mobile phase B contained 0.1% formic acid in 80% acetonitrile. Non-targeted analysis was performed in a data-dependent acquisition (DDA) mode, fragmenting the ten most abundant, multiply charged ions, with dynamic exclusion time set to 60 s. Each sample was measured in three analytical replicates. A full list of instrument parameters is given in Supplementary Table 1.

### Protein identification and data analysis

Mass spectrometric data from each LC-MS run were analyzed using the MaxQuant software^49,50^ (Version 1.6.14). The identification of proteins was performed using the MaxQuant-implemented Andromeda search engine against a reference Homo sapiens proteome. Initial maximum precursor and fragment mass deviations were set to 7 ppm and 0.5 Da, respectively. Variable modifications (methionine oxidation and N-terminal acetylation) and fixed modifications (cysteine carbamidomethylation) were set for the search and trypsin with a maximum of three missed cleavages was chosen for searching. The minimum peptide length was set to 7 amino acids and false discovery rate (FDR) for peptide and protein identification was set to 0.01.

### KEGG pathway enrichment

KEGG pathway^51–53^ analysis enrichment was conducted using STRING (https://string-db.org/), a widely used bioinformatics tool for exploring functional interactions between proteins^54^.

## Results

### M0 is the most adequate dTHP-1 phenotype for assessing nanofiber toxicity

Two initial experiments were conducted to select both the differentiation duration and the LPS concentration needed to induce an M1 phenotype that is sensitive to material exposure. This was assessed using the LDH assay and measurements of TNF-α levels. Supplementary Fig. 1 shows the differentiation results focusing on the initial PMA concentration and differentiation time, while Supplementary Fig. 2 presents findings on the inclusion of a 24-hour rest period in PMA-free media and the optimal LPS concentration. Optimal M1 differentiation was achieved with a 24-hour differentiation period using 100 nM PMA, without a rest period, followed by 20 ng/mL IFN-γ and 200 ng/mL LPS for 24 h, consistent with the conditions utilized by Mirata et al.^55^. The next step of our study was to determine by testing cytotoxicity and pro-inflammatory marker release, which of the dTHP-1 phenotypes—M0, M1, or M2—is most sensitive to the morphological variations of different carbon-based NMs, specifically Printex-90, NM-400, and Mitsui-7-JRCNM40011a.

**Fig. 1:**
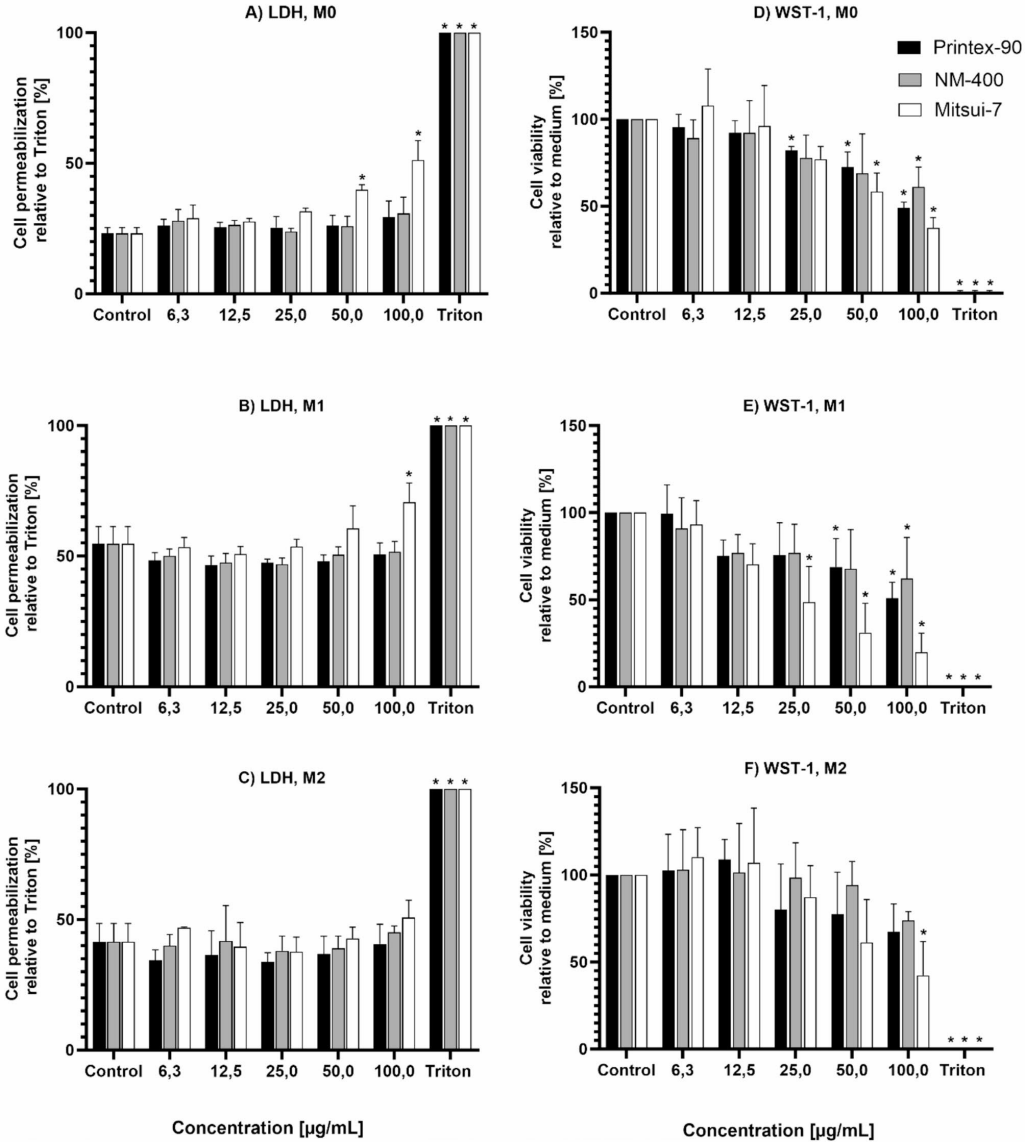
Cytotoxicity assessment in M0, M1, and M2 dTHP-1 macrophage phenotypes exposed for 24 h to various concentrations of carbon-based NMs, Printex-90, NM-400 and Mitsui-7-JRCNM40011a. (**A-C)** Results from the LDH assay. Data are presented as a percentage of LDH release relative to the maximum release induced by lysis with 1% Triton-X 100 (set as 100%). (**D-F)** WST-1 assay results showing metabolic activity expressed as a percentage relative to the untreated control (set as 100%). Each bar denotes the mean ± SD from three independent experiments (*n* = 3). “*” indicates a statistically significant difference from the control group by the one-way ANOVA, followed by Dunnett’s multiple comparisons test with *p* < 0.05.

Cell toxicity was assessed using the LDH and WST-1 assays. Figure 1 (A-C), shows the percentage of LDH release following exposure to different concentrations of Printex-90, NM-400, and Mitsui-7-JRCNM40011a, normalized to the complete cell lysis with 1% Triton-X. Results indicate a concentration-dependent effect of Mitsui-7-JRCNM40011a on the LDH release for M0 and M1 dTHP-1 phenotypes, with significant LDH release observed at starting concentrations of 50 µg/mL and 100 µg/mL for M0 and M1, respectively. No significant LDH release was observed for Printex-90 and NM-400 in any assessed phenotypes. Notably, the baseline for the M1 dTHP-1 phenotype was exceptionally high, indicating that 55% of the cells are non-viable even in the absence of any treatment. The M2 dTHP-1 phenotype, also exhibited a high LDH baseline of 40% and furthermore, appeared to be unaffected by any of the NMs evaluated.

The metabolic activity of the exposed cells, relative to an untreated control, was assessed by means of the WST-1 assay, as shown in Fig. 1 (D-F). Similar to the LDH assay, Mitsui-7-JRCNM40011a showed impairment of metabolic activity for the M0 and M1 dTHP-1 phenotypes at concentrations 50 µg/mL and 25 µg/mL, respectively. However, Printex-90 showed a similar dose-response effect to Mitsui-7-JRCNM40011a for the M0 and M1 dTHP-1 phenotypes, starting at 25 µg/mL and 50 µg/mL, respectively. NM-400 only affected the M0 phenotype at the highest concentration evaluated, 100 µg/mL. In contrast to the LDH measurement, the M2 dTHP-1 phenotype was only affected by Mitsui-7-JRCNM40011a at the highest evaluated concentration, 100 µg/mL. Overall, the results presented in Fig. 1 show that dose-dependent cytotoxic effects vary amongst the different dTHP-1 macrophage phenotypes, indicating that the macrophage activation state influences their susceptibility to the cytotoxic effects of the NMs. Notably, M0 dTHP-1 macrophages showed the most promising response.

The pro-inflammatory response of dTHP-1 macrophage phenotypes, when exposed to the carbon-based NMs, was evaluated by measuring their TNF-α release. The response was compared to LPS treatment, which serves as a pro-inflammatory positive control. Of the three phenotypes evaluated, only the M0 dTHP-1 phenotype showed a significant response, and only at the highest concentration evaluated, 100 µg/ml with Mitsui-7-JRCNM40011a. The M1 dTHP-1 phenotype showed no response to tested materials, while M2 phenotype showed minimal inflammatory capacity.

**Fig. 2:**
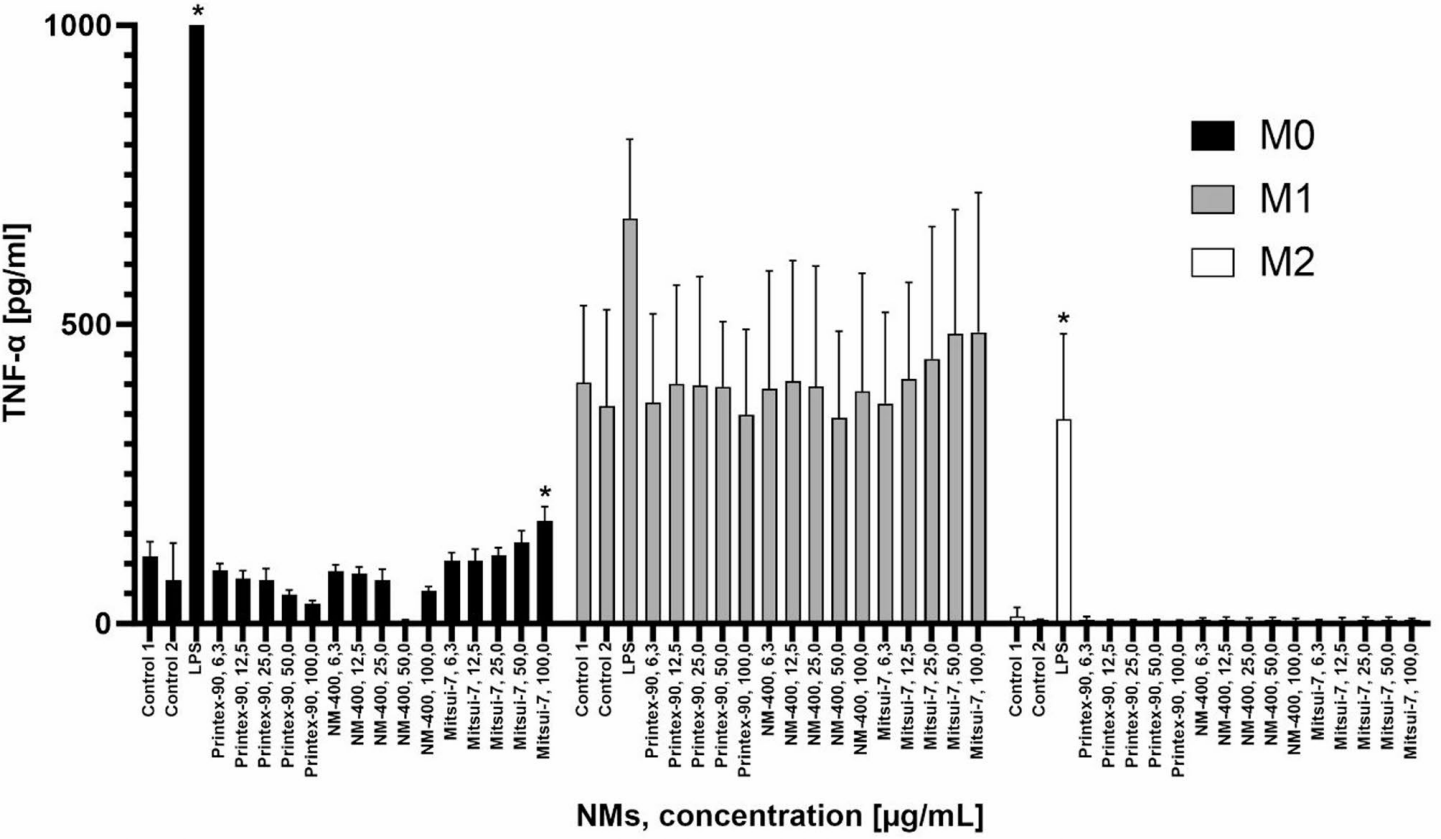
TNF-α levels in the M0, M1 and M2 dTHP-1 macrophage phenotypes exposed for 24 h to various concentrations of Printex-90, NM-400 and Mitsui-7-JRCNM40011a, or 1 µg/mL of LPS. Each bar represents the mean ± SD of three independent experiments (*n* = 3). “*” indicates a statistically significant difference from the control group, of each respective phenotype, by the one-way ANOVA, followed by Dunnett’s multiple comparisons test with *p* < 0.05.

Taken together, the results presented in Fig. 2 show that TNF-α release varies amongst the different dTHP-1 macrophage phenotypes. Furthermore, due to the low response after NM exposure, the results suggest that the TNF-α measurement may not be a reliable indicator of an inflammatory response that can be used to discern NM morphological effects.

Taken together, of all the dTHP-1 phenotypes evaluated for cytotoxicity and inflammatory response (Table 2), the M0 phenotype appears to be the most sensitive model for further detailed investigation. The M1 phenotype was excluded from further analysis due to the extremely high baseline toxicity seen in the LDH assay, which was already 55% under control conditions indicating significant impairment of cell viability even without treatment. Furthermore, despite the induction of inflammation, there was no response to any NM exposure. Similarly, the M2 phenotype exhibited high LDH baseline levels and as expected from its anti-inflammatory nature, exhibited no NM-induced inflammatory response. In light of these findings, both the M1 and M2 phenotypes were excluded and the M0 dTHP-1 phenotype was used further to investigate the toxicity of the different morphologies of the carbon-based NMs.

**Table 2:** Overview of the response across the different dTHP-1 phenotypes to the different morphologies of the carbon-based nms, highlighting their different sensitivities.

	Assay	M0	M1	M2
Baseline Toxicity	LDH	Acceptable (23%)	Too high (55%)	Too high (40%)
Dose - Response	LDH	Yes	–	–
	WST-1	Yes	Yes	–
	TNF-α	Possible*	–	–

The comparison is based on two cytotoxicity assays, LDH and WST-1, along with a TNF-α release assay as an inflammatory readout. *A positive trend was observed, however, a significant response relative to the control was only observed at the highest concentration tested.

### Rigid Mitsui-7 shows the stronger responses in the toxicological screening

Subsequently, we evaluated the morphological toxicity of carbon-based NMs using both acellular and cellular assays. The acellular assay was designed to detect the inherent reactivity of the NMs. For the cellular assays, we employed M0 dTHP-1 macrophages to assess oxidative damage, lysosomal integrity, and for an in-depth proteomic profiling.

**Fig. 3:**
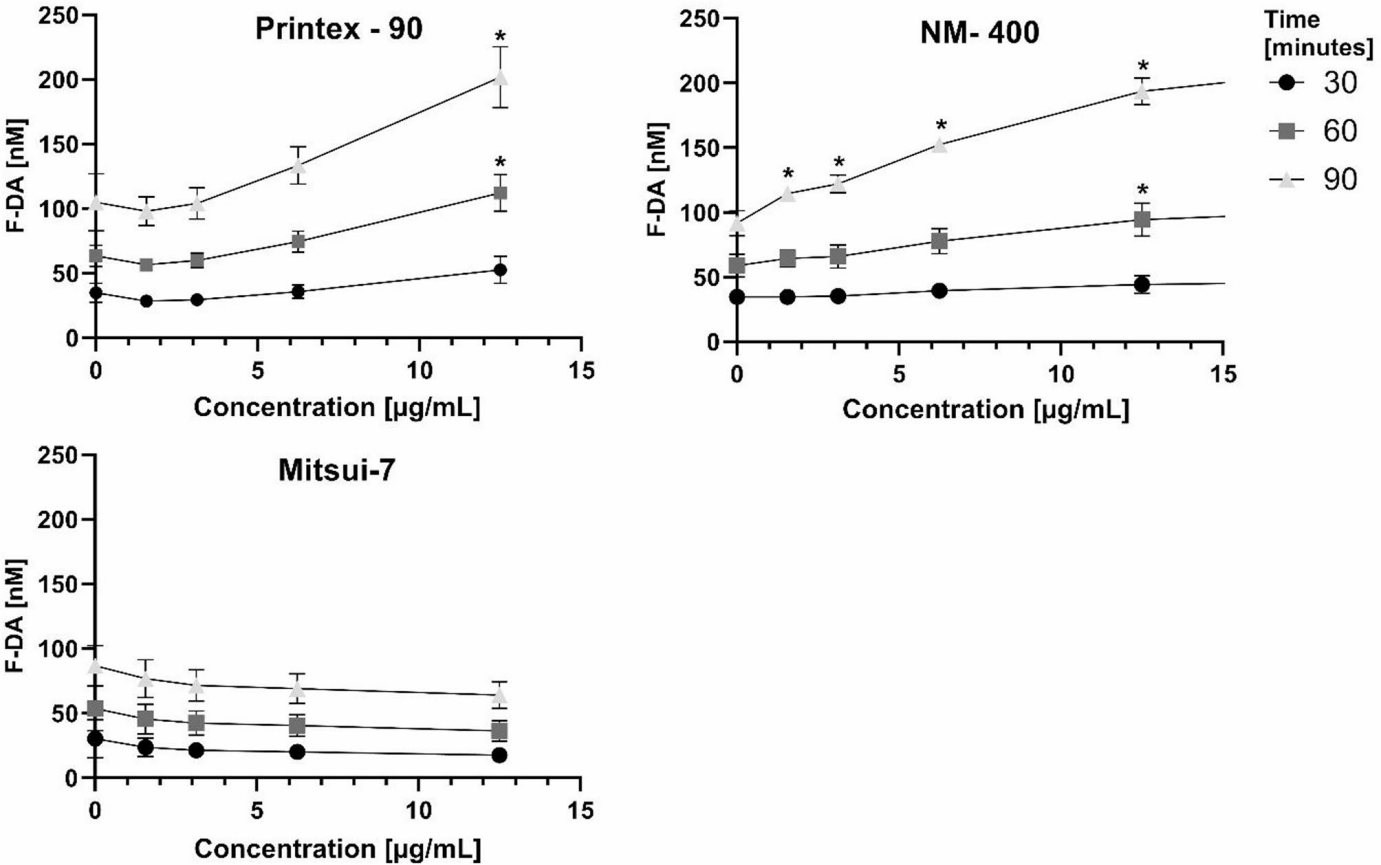
Reactivity assessment of Printex-90, NM-400, and Mitsui-7-JRCNM40011a. Data is presented as the concentration of fluorescein diacetate (F-DA) in nM, achieved by normalizing the arbitrary fluorescence unit’s readout of the assay to a F-DA standard curve. Each line represents the assay readout measured across three different timepoints; 30 (⬤), 60 (■) and 90 (▲) mins. Each datapoint denotes the mean ± SD from three independent experiments (*n* = 3). “*” indicates a statistically significant difference from the control group by the one-way ANOVA, followed by Dunnett’s multiple comparisons test with *p* < 0.05.

The DCFH_2−_DA assay was employed to determine the inherent reactivity of the different NMs by measuring the production of ROS in an acellular setup over various time intervals. Figure 3 shows the reactivity assessment of Printex-90, NM-400 and Mitsui-7-JRCNM40011a. Mitsui-7-JRCNM40011a does not show acellular activity; instead, there is a slight decrease in dichlorofluorescein (DCF) fluorescence at higher concentrations, which may suggest antioxidative properties or a scavenging effect of the material. In contrast, results suggest a time and concentration-dependent increase in reactivity for Printex-90 and NM-400, which is significant for both materials at 12,5 µg/mL.

Comparing these results with the cytotoxicity measurements based on LDH release shown in Fig. 1A-C, where no significant LDH release was observed at NM concentrations up to 12.5 µg/mL in any of the assessed dTHP-1 phenotypes, it can be concluded that the increased reactivity of the evaluated NMs, although notable, is insufficient to compromise the integrity of the cellular membrane. Similarly, the LDH release detected in Mitsui-7-JRCNM40011a at concentrations starting from 25 µg/mL does not correlate with the material reactivity indicating that other factors may be contributing to the cellular toxicity relative to membrane permeabilization.

**Fig. 4:**
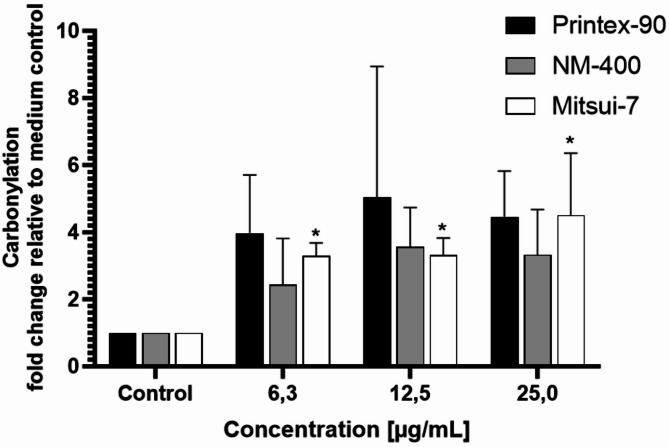
Oxidative protein damage assessment in M0 dTHP-1 macrophages exposed to different carbon-based NMs for 24 h, measured by protein carbonylation quantification. Each bar denotes the mean ± SD from three independent experiments (*n* = 3). “*” indicates a statistically significant difference from the control group by the one-way ANOVA, followed by Dunnett’s multiple comparisons test with *p* < 0.05.

Protein carbonylation is an indicator of oxidative protein damage and is a recognized marker of cellular oxidative stress^56^. Figure 4 shows the levels of protein carbonylation in the M0 dTHP-1 phenotype exposed to the three different carbon-based NMs. At all tested concentrations from 6.3 µg/mL to 25 µg/mL, all NMs show an increase in protein carbonylation, although these values are only statistically significant for Mitsui-7.

**Fig. 5:**
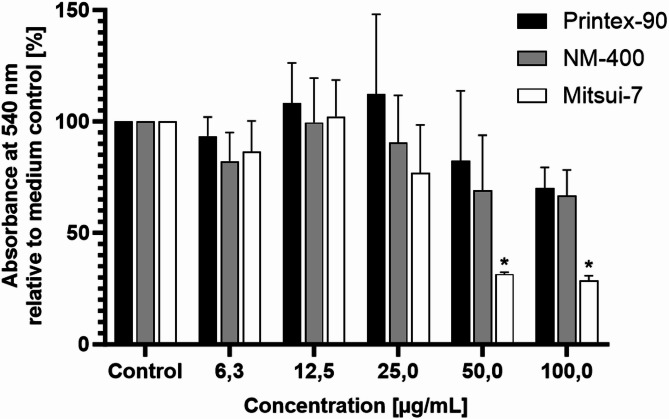
NR assay activity in dTHP-1 M0 macrophages exposed to various concentrations of carbon-based NMs for 24 h. Data is presented as a percentage of measured NR absorbance at 540 nm relative to the untreated control (set as 100%). Each bar represents the mean ± SD of three independent experiments (*n* = 3). “*” indicates a statistically significant difference from the control group by the one-way ANOVA, followed by Dunnett’s multiple comparisons test with *p* < 0.05.

The NR assay is conventionally employed for the assessment of cytotoxicity; however, in our study it is utilized as an indirect readout for lysosomal integrity because it can reflect changes in lysosomal permeability. While a decline in the assay readout indicates potential lysosomal damage and/or cytotoxic effects, a comparison among the three carbon-based NMs, as shown in Fig. 5, reveals that only Mitsui-7-JRCNM40011a causes a pronounced decrease at concentrations ≥ 50 µg/mL withPrintex-90 and NM-400 having no statistically significant effects.

### Proteomic profiling indicates lysosomal disruption

Proteomic profiling was performed to gain insight into the molecular mechanisms affected by the different morphologies of the carbon-based NMs. We used two different concentrations of 25 µg/mL and 50 µg/mL of the NMs to treat M0 dTHP-1 cells during 24 h. The complete list of detected proteins is shown in Supplementary Tables 2 to 11, for treatments with NM concentrations of 25 µg/mL and 50 µg/mL, respectively.

Summarized, at a concentration of 25 µg/mL and 50 µg/mL, 3217 and 3226 total proteins were detected, respectively. Exposure to Printex-90 at concentrations of 25 µg/mL and 50 µg/mL resulted in 43 and 134 significantly altered proteins; respectively. In contrast, exposure to NM-400 at the same concentrations resulted in 15 and 65 significantly altered proteins, respectively. Mitsui-7-JRCNM40011a accounts for the highest number of significantly altered proteins, affecting 257 at a concentration of 25 µg/mL, and 777 proteins at a concentration of 50 µg/mL.

**Fig. 6:**
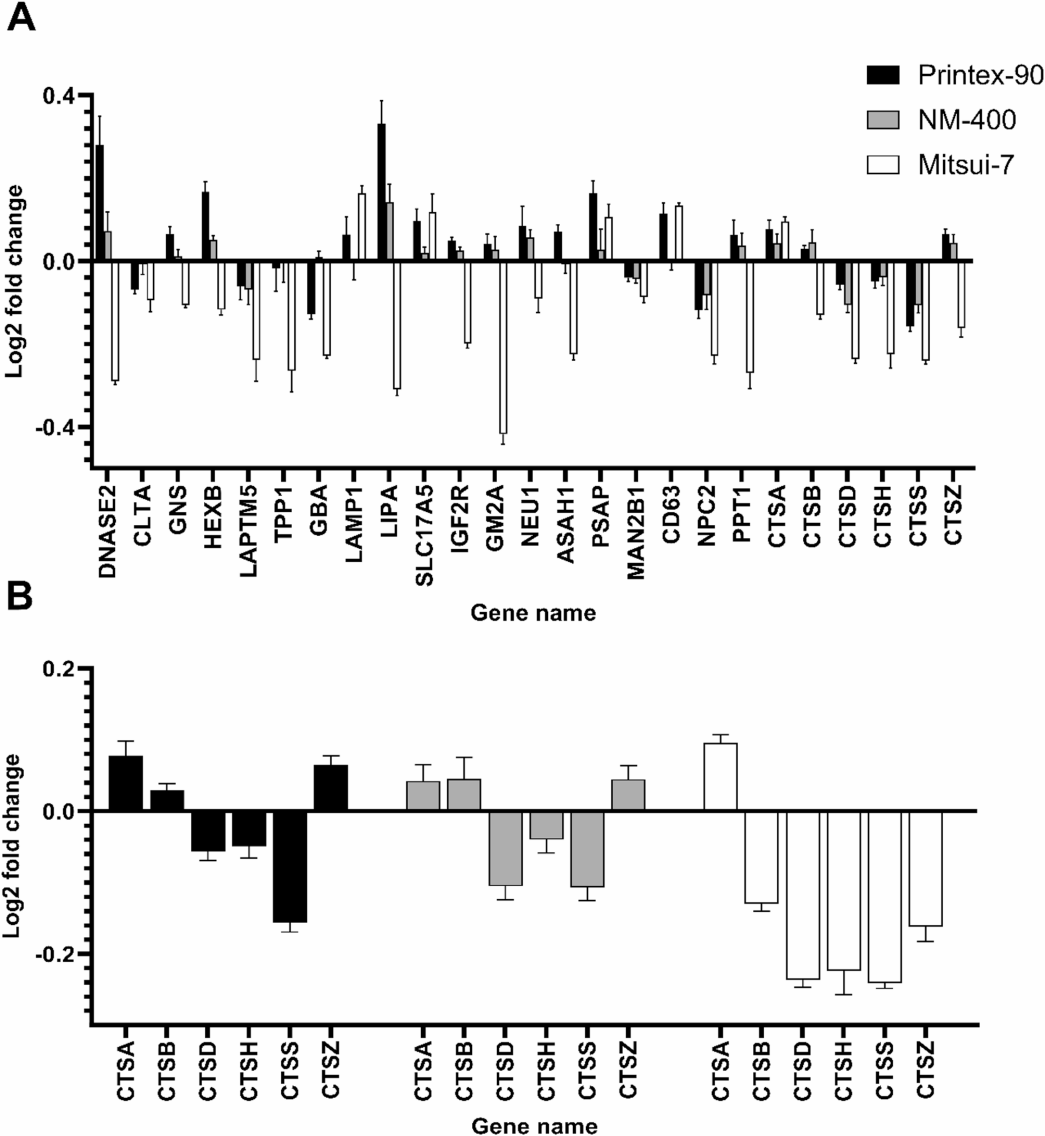
(**A**) Level of significantly altered lysosomal proteins as detected by proteomics following exposure of M0 dTHP-1 cells to 50 µg/mL of the different NMs. (**B**) Detailed comparison of significantly altered cathepsins.

Subsequently, we subjected the list of significantly altered proteins to an enrichment analysis using the STRING database to identify altered pathways (Table 3). As indicated by the enrichment analysis, Printex-90 affected only the cholesterol metabolism KEGG pathways in a dose-dependent manner. At 50 µg/mL, additional KEGG pathways including ribosome, spliceosome, and DNA replication were also affected. Due to the low number of significantly altered proteins for NM-400, no altered KEGG pathway was detected by our analysis. Mitsui-7-JRCNM40011a appeared to be the only material that affected the lysosomal KEGG pathway and notably, this effect occurred in a dose-dependent manner, supporting the screening results from the NR assay. Additionally, from the 777 proteins altered by exposure to 50 µg/mL of Mitsui-7-JRCNM40011a, pathways associated with shigellosis, Parkinson’s disease, metabolic pathways, carbon metabolism, and protein processing in the endoplasmic reticulum were affected. Notably, the latter three pathways are related to the maintenance of cellular homeostasis. 25 of the 777 proteins are associated with the lysosomal KEGG pathway and interestingly, the vast majority of these proteins are down-regulated (Fig. 6-A), indicating that their levels are significantly lower compared to control conditions.

Notably, six of the lysosomal proteins are cathepsins, of which five are down-regulated after Mitsui-7-JRCNM40011a exposure, as indicated by Fig. 6-B. Cathepsins are a group of protease enzymes primarily found in lysosomes, involved in protein degradation and various cellular processes. Supplementary Table 12 includes a complete list of the 25 lysosomal proteins significantly altered by Mitsui-7-JRCNM40011a, showing their relative levels to the control under the different evaluated conditions.

**Table 3:** KEGG pathway enrichment analysis of significantly altered proteins as identified by proteomic measurements for Printex-90 and Mitsui-7.

KEGG Pathway	25 µg/mL	50 µg/mL
**Printex-90**		
Cholesterol metabolism	4 of 48 (0.0016)	6 of 48 (0.00026)
Ribosome	-	10 of 131 (1.20e-05)
Spliceosome	-	7 of 132 (0.0034)
DNA replication	-	4 of 36 (0.0096)
**Mitsui-7**		
Lysosome	14 of 125 (1.03e-06)	25 of 125 (2.47e-08)
Shigellosis	13 of 218 (0.0015)	-
Metabolic pathways	-	133 of 1435 (7.20e-17)
Carbon metabolism	-	34 of 116 (2.65e-15)
Protein processing in ER	-	30 of 163 (4.39e-09)
Parkinson disease	-	36 of 236 (4.40e-09)

Numbers indicate how many proteins were detected as significantly altered out of the total of proteins belonging to that particular KEGG pathway. Numbers in brackets represent the FDR calculated for the enrichment of that pathway. Only the top 5 KEGG pathways are displayed if the FDR < 0.01.

## Discussion

Risk assessment relies heavily on animal testing but to align with the European Commission efforts to enable the transition from animal testing to alternative approaches, NAMs are needed. The aim of this study was to use NAMs to move towards a harmonized testing strategy, in particular by applying in vitro assays for assessing the pathogenicity of NFs. It is crucial to recognize that the provided assays in the presented study contribute to advancing the hazard assessment framework evaluating NF toxicity. In addition to the cellular response, assessment of the morphology and agglomeration state, as well as the assessment of biopersistence must be further considered, as they play a key role in determining NF pathogenicity. For instance, biopersistence refers to both the physicochemical stability/durability of a fiber in the body (such as its solubility or dissolution rate) and the efficiency of biological clearance mechanisms, particularly those mediated by macrophages^57^. However, the assays proposed in the present work do not allow the necessary time horizons to adequately account for the dissolution behavior and solubility of the NFs, which should be evaluated separately.

To develop NAMs for assessing the hazard of NFs, it is essential to address open questions such as the suitability of the cell model and whether proposed assays are sufficiently reliable. Moreover, we investigated further relevant mechanistic processes using an advanced tandem-mass tag (TMT) labelling protocol^39^ for proteome profiling. Although this analysis is not intended as an assay per se within the test strategy, it was performed to obtain mechanistic information on NF toxicity. It thereby supports the substantiation of AOPs and further development of NAMs, which may be subsequently incorporated into an overall testing strategy.

Carbon-based NMs of different morphologies were used in our study to address these questions with regard to a fiber test strategy. MWCNTs are among the most extensively studied NFs with a substantial amount of existing knowledge on NF toxicity both in vitro and in vivo^58,59^. MWCNTs can exists as rigid and tangled forms, with a proposed bending threshold of 30 nm in diameter^11^. While Mitsui-7-JRCNM40011a surpasses this threshold and is considered a rigid fiber, NM-400 fibers fall below this limit and are considered non-rigid NF. In addition, particulate Printex-90 is also applied for testing. dTHP-1 cells were used in our study as they are a common cell model for studying macrophage responses in immunology, infectious diseases, inflammatory diseases, and other areas. This choice is partly due to the limited number of alternatives.

### The dTHP-1 M0 phenotype provides sensitive and reliable readout for testing fiber toxicity

The physiological response of macrophages to inhaled NFs is a multi-step process which employs multiple phenotypes across the inflammatory cascade. Therefore, as a first step, we undertook a critical evaluation of the suitability of different dTHP-1 phenotypes, which have proven their adequacy to test the toxicity of asbestos fibers^28^.

Having optimized the differentiation and polarization conditions to obtain the various dTHP-1 phenotypes, we determined which phenotype was more sensitive in discerning the effect of the different morphologies of the carbon-based NMs. We considered measurements of cytotoxicity as assessed by the LDH and WST assays, which address cellular membrane permeability and metabolic activity, respectively, and pro-inflammatory response by measuring TNF-α release. The M0 phenotype displayed both a cytotoxic and pro-inflammatory response to Mitsui-7-JRCNM40011a, while the other two NMs only caused a decrease in the WST-1 assay readout, suggesting an impact on metabolic activity without inducing cell permeability, as evidenced by the LDH assay.

According to the TNF-α levels, the M1 phenotype exhibited an intense pro-inflammatory state relative to the other dTHP-1 phenotypes, likely inherent to the polarization process. However, no differences in the intensity of the pro-inflammatory response after NM exposure were observed for any of the NM tested. Previous omic investigations support our finding by showing that the polarization to M1 in both dTHP-1 cells and mouse RAW264.7 macrophages resulted in up-regulation of the TNF-α as well as NF-κB signaling pathways relative to M2^60^. Whilst it is arguable whether the release of TNF-α is the optimal indicator of inflammation in dTHP-1 cells, a compelling rationale for excluding the M1 phenotype from further analysis arose from the exceptionally high baseline detected in the LDH assay. This suggests that the additional polarization process required to obtain the M1 dTHP-1 phenotype had a cytotoxic effect, resulting in membrane permeability in over half of control cells. We therefore, recommend to report LDH data of control and treated cells, normalized to a positive control for which complete cellular permeabilization is achieved, such as exposure to Triton-X.

The M2 phenotype displayed a complete lack of pro-inflammatory response consistent with its anti-inflammatory characteristics, in addition to the excessively high baseline LDH level.

Altogether, the M0 dTHP-1 phenotype was selected for further investigation of the toxicity of various morphologies of carbon-based NMs.

### Mitsui-7 shows the most dominant response in screening assays

Given that lysosomal perturbation is implicated as part of frustrated phagocytosis^61–63^, it became paramount to assess lysosomal integrity utilizing the NR assay. A decline in the NR assay readout suggested lysosomal damage at concentrations ≥ 50 µg/mL in M0 dTHP-1 cells exposed to Mitsui-7-JRCNM40011a, contrary to the other NMs, which showed no significant effects. This result for Mitsui-7-JRCNM40011a is consistent with findings in the literature and for a comparable long rigid MWCNT, such as NM-401, known to caused lysosomal membrane permeabilization (LMP)^64,65^. Increased levels of LMP was also reported for NF of TiO_2_^63^, CeO_2_^66^, anodic Al^67^, and Ag^68^, but not for spherical NPs of the same chemical composition in vitro, possibly due to effective compartmentalization into the lysosomes^65,69^. Similar observation correlate with in vivo findings^63,70^. As frustrated phagocytosis cannot be readily quantified, lysosomal damage could be a suitable marker of morphology-dependent toxicity indicating a cell undergoing frustrated phagocytosis^24^.

Following frustrated phagocytosis of bio-persistent fibers, increased ROS are generated due to the oxidative bursts in macrophages, which can be a significant source of oxidative stress to surrounding cells^71^. Oxidative stress therefore is a KE in fiber toxicity AOPs and it can subsequently lead to secondary genotoxicity, including oxidative lesions to DNA. Additionally, it is well documented that Printex-90^72^ and Mitsui-7^73,74^ induce the production of ROS as part of their toxicity mechanism. Therefore, we investigated different assays to distinguish between the inherent oxidative capacity of the NMs and their cell-associated effects in macrophages. To evaluate the inherent oxidative capacity of the NMs, we conducted the DCFH_2_-DA assay under acellular conditions, while a carbonylation assay was used to detect ROS formation in M0 dTHP-1 cells. Printex-90 and NM-400 exhibited inherent oxidative capacity, unlike Mitsui-7-JRCNM40011a, which interestingly not only did not exhibit ROS production but also presented a slight decrease in DCF under acellular conditions. This may be due to the intrinsic scavenging capacity of MWCNTs, meaning they can adsorb ROS at their surface^75^ which has been suggested to be related to the amount and nature of structural defects in the MWCNTs^76^. However, all NMs showed the ability to oxidize proteins, as evidenced by increased protein carbonylation; however, values were only significantly increased for Mitsui-7, due to the high standard deviation observed for both Printex-90 and NM-400. Notably, Mitsui-7-JRCNM40011a induced protein carbonylation within cells despite lacking inherent oxidative capacity, implying a more intricate interaction with cellular components, which leads to cellular ROS generation compared to the other NMs. These results indicate that macrophages undergo oxidative stress when treated with Mitsui-7-JRCNM40011a, probably as a consequence of frustrated phagocytosis.

### Proteomic profiling to unravel mechanisms of NF toxicity

To complement the in vitro toxicological screening approaches used, which are limited to the assessment of acute toxicity, proteomics was used to gain a more in-depth knowledge of altered cellular pathways. This analysis confirmed the results of the NR assay and showed that Mitsui-7-JRCNM40011a induced lysosomal disruption to a greater extent than the other NMs evaluated. Of the 777 significantly altered proteins, 25 proteins were found to be associated with the lysosomal KEGG pathway at Mitsui-7.

A thorough analysis of lysosomal protein levels revealed that the majority of these proteins showed lower abundance levels in the cells treated with Mitsui-7-JRCNM40011a. Of particular interest is the finding that among the 25 lysosomal proteins significantly altered, six of them are cathepsins, five of which were down-regulated in response to Mitsui-7-JRCNM40011a exposure. This finding aligns with previous studies indicating that the release of cathepsin B into the supernatant is a consequence of fiber toxicity^27,64,65^. Considering this result, the decreased levels of cathepsins observed in our proteomic analysis, may be attributed to increased LMP due to frustrated phagocytosis leading to the leakage of lysosomal proteins into the extracellular milieu. Studies investigating NF-related effects are often limited to cathepsin B release^24,65,68^ and only a limited number of publications aimed to quantify other cathepsins for this purpose^61^. However, multiple cathepsins have been associated with LMP and subsequent inflammasome activation^77^. Therefore, measuring multiple cathepsins and quantifying these with targeted approaches both in cell lysates and supernatants may provide a more comprehensive overview and robust readout for NF toxicity. Notably, dTHP-1 cells may pose a challenge for conducting secretomics experiments due to the necessity to include FCS for their culture and viability^78,79^. FCS in the CCM compromises in-depth proteomic analysis of the secretome, as secreted proteins may be overshadowed to their low abundance^80^. Alternative cell line such as NR8383 rat macrophages, which can be exposed under FCS-free conditions, have shown their potential for predictive inhalation toxicity for NMs^81^.

In summary, since frustrated phagocytosis cannot be directly measured in vitro, our observation of multiple cathepsin alterations and quantifying these with targeted proteomic analysis, may serve as viable biological markers indicative of morphology-driven fiber toxicity.

## Conclusion

This study aims to advance a harmonized testing strategy for assessing NF pathogenicity using in vitro NAMs. It addresses key issues like cell model suitability and assay reliability using dTHP-1 cells to evaluate the sensitivity of different phenotypes (M0, M1, M2) to morphological variations of carbon-based NMs. dTHP-1 cells proved to be an adequate model for evaluating NF toxicity, with the M0 phenotype identified as the most suitable. An array of screening assays highlighted that Mitsui-7-JRCNM40011a causes cytotoxicity, pro-inflammatory response, oxidative stress and lysosomal disruption. Proteomic profiling confirmed this last finding, and evidenced a decrease in the level of lysosomal proteins, particularly cathepsins, which may serve as potential biomarkers of frustrated phagocytosis and NF toxicity. This comprehensive approach underscores the utility of proteomics alongside traditional assays for a deeper mechanistic understanding. Moreover, it enhances the understanding of NF toxicity, contributing to the development of a reliable in vitro testing strategy by the identification of putative biomarkers of NF toxicity.

## Supplementary Information

Below is the link to the electronic supplementary material.

Supplementary Material 1

Supplementary Material 2

Supplementary Material 3

Supplementary Material 4

Supplementary Material 5

Supplementary Material 6

Supplementary Material 7

Supplementary Material 8

Supplementary Material 9

Supplementary Material 10

Supplementary Material 11

Supplementary Material 12

Supplementary Material 13

Supplementary Material 14

## Data Availability

The datasets used and analyzed during the current study are available from the corresponding author on reasonable request.

## Abbreviations

CCM: Cell culture medium
FPP: Fiber pathogenicity paradigm
FDR: False discovery rate
LDH: Lactate dehydrogenase
LPS: Lipopolysaccharide
MS: Mass spectrometry
NF: Nanofibers
NM: Nanomaterial
NR: Neutral red
ROS: Reactive oxygen species
TFA: Trifluoroacetic acid
WST: Water soluble tetrazolium
