# Supplementary material for: Towards a harmonized testing strategy for nanofibers by integrating toxicological screening and proteomic profiling
Source: Sci Rep. 2025 Sep 12;15:32430. doi: 10.1038/s41598-025-15423-9 (PMC12432259; doi:10.1038/s41598-025-15423-9)
Supplement: Supplementary file 2 — Supplementary Material 2 [file 41598_2025_15423_MOESM2_ESM.docx]

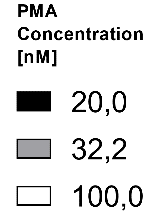
**Supplementary Figures and Figure Legends**

**Supplementary Figure 1:** LDH cytotoxicity assessment in M0 (A-B), M1 (C-D), and M2 (E-F) dTHP-1 macrophage phenotypes, exposed for 24h to various concentrations of Mitsui-7- JRCNM40011a. The different cell models were differentiated with either 20 (black bars), 32,2 (grey bars) or 100,0 (white bars) nM PMA for 24h (A, C, D) or 48h (B, D, E). Data are presented as a percentage of LDH release relative to the maximum release induced by lysis with 1% Triton-X 100 (set as 100%). Each bar denotes the mean ± SD from three independent experiments (n=3). "*" indicates a statistically significant difference from the control group by the one-way ANOVA, followed by Dunnett’s multiple comparisons test with p<0.05.


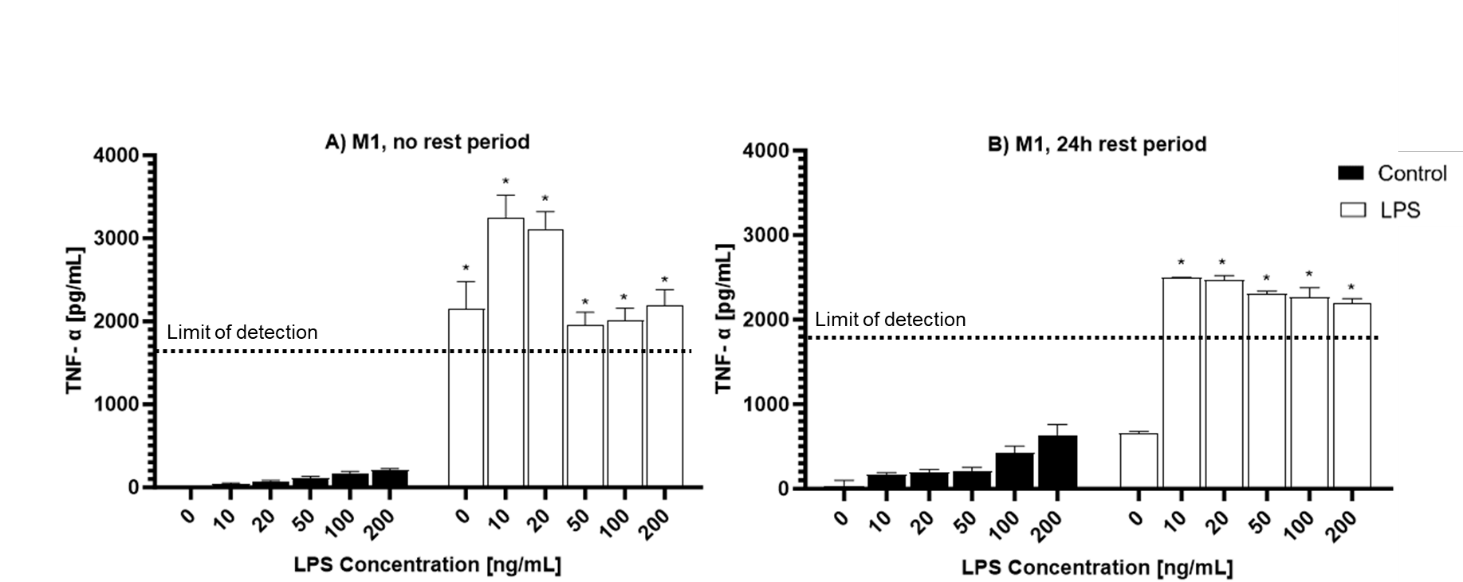


**Supplementary Figure 2:** Various M1 phenotype models were obtained using M0 dTHP-1 (differentiated with 100 nM PMA for 24h), rested with (B) or without (A) a rest period of 24h in PMA CCM, and then further polarized with 20 ng/mL of IFN-γ and various LPS concentrations (x-axis) as investigated. TNF-α levels in these various models were assessed after exposure to 24h with CCM which represents control conditions (black bars), 100 µg/mL of Mitsui-7- JRCNM40011a (grey bars), or 1µg/mL of LPS (white bars). Each bar represents the mean ± SD of three independent experiments (n=3). "*" indicates a statistically significant difference for the Mitsui-7- JRCNM40011a or LPS exposure, when compared to the control conditions of the same polarisation conditions, by the one-way ANOVA, followed by Dunnett’s multiple comparisons test with p<0.05. The dashed line indicates the threshold for TNF-α levels, beyond which the corresponding absorbance values exceeding the instrument's detection range.
