# Supplementary material for: Towards a harmonized testing strategy for nanofibers by integrating toxicological screening and proteomic profiling
Source: Sci Rep. 2025 Sep 12;15:32430. doi: 10.1038/s41598-025-15423-9 (PMC12432259; doi:10.1038/s41598-025-15423-9)
Supplement: Supplementary file 3 — Supplementary Material 3 [file 41598_2025_15423_MOESM3_ESM.docx]

**Supplementary Material**

**Towards a Harmonized Testing Strategy for Nanofibers: Integrating Toxicological Screening and Proteomic Profiling**

**Rico Ledwith^1,3^, Verónica I. Dumit^1^, Tobias Stobernack^1^, Victor Alcolea-Rodriguez^1,2^, Antje Bergert^1^, Doreen Wittke^1^, Andrea Haase^1,3^ⱡ*, Mario Pink^1^ⱡ***

**^1^**German Federal Institute for Risk Assessment (BfR), Department of Chemical and Product Safety, Berlin, Germany; Institute of Pharmacy, Freie Universität Berlin, Berlin, Germany.

**^2^**Spanish National Research Council (CSIC), Institute for Catalysis and Petroleum Chemistry, Madrid, Spain

**^3^**Freie Universität Berlin, Institute of Pharmacy, Berlin, Germany

ⱡAuthors contributed equally

*****Corresponding authors: [Mario.Pink@bfr.bund.de](mailto:Mario.Pink@bfr.bund.de) and Andrea.Haase@bfr.bund.de

**Supplementary Information:**

**Supplementary Data:** Supplementary Figure 1 and Supplementary Figure 2.

**Supplementary Table 1:** LC-MS parameters.

**Supplementary Table 2:** TMT 6-plex reporter ion intensities of all detected proteins in dTHP-1 M0 cells untreated or incubated with 25 µg/mL of Printex-90, NM-400, Mitsui-7.

**Supplementary Table 3:** TMT 6-plex reporter ion intensities of all filtered proteins in dTHP-1 M0 cells untreated or incubated with 25 µg/mL of Printex-90, NM-400, Mitsui-7.

**Supplementary Table 4:** Significantly detected proteins for 25 µg/mL Printex-90 relative to control conditions.

**Supplementary Table 5:** Significantly detected proteins for 25 µg/mL NM-400 relative to control conditions

**Supplementary Table 6:** Significantly detected proteins for 25 µg/mL Mitsui-7 relative to control conditions

**Supplementary Table 7:** TMT 6-plex reporter ion intensities of all detected proteins in dTHP-1 M0 cells untreated or incubated with 50 µg/mL of Printex-90, NM-400, Mitsui-7

**Supplementary Table 8:** TMT 6-plex reporter ion intensities of all filtered proteins in dTHP-1 M0 cells untreated or incubated with 50 µg/mL of Printex-90, NM-400, Mitsui-7

**Supplementary Table 9:** Significantly detected proteins for 50 µg/mL Printex-90 relative to control conditions

**Supplementary Table 10**: Significantly detected proteins for 50 µg/mL NM-400 relative to control conditions

**Supplementary Table 11**: Significantly detected proteins for 50 µg/mL Mitsui-7 relative to control conditions

**Supplementary Table 12:** List of the 25 lysosomal proteins significantly altered by 50 µg/mL Mitsui-7
